# Quantitative [^18^F]FDOPA PET/CT for the characterization of biochemical phenotypes in paraganglioma and pheochromocytoma

**DOI:** 10.1186/s41824-025-00275-3

**Published:** 2025-11-24

**Authors:** Paul Dahlmann, Meike Onkes, Sophie Kunte, Matthias K. Auer, Ulrike Disko, Katharina Wang, Christian Lottspeich, Isabel Stüfchen, Júnia R.O.L. Schweizer, Nabeel Mansour, Matthias Brendel, Christine Schmid-Tannwald, Alessa Fischer, Svenja Nölting, Christoph J. Auernhammer, Martin Reincke, Martin Bidlingmaier, Rudolf A. Werner, Matthias Kroiss, Friederike Völter

**Affiliations:** 1 Department of Nuclear Medicine, LMU University Hospital, LMU Munich, 81377 Munich, Germany; 2 Department of Medicine IV, Endocrinology, Diabetes and Metabolism, LMU University Hospital, LMU Munich, 80336 Munich, Germany; 3 Department of Radiology, LMU University Hospital, LMU Munich, 81377 Munich, Germany; 4 Department of Endocrinology, Diabetology and Clinical Nutrition, University Hospital Zurich (USZ) and University of Zurich (UZH), Zurich, 8091 Switzerland; 5 The Russell H Morgan Department of Radiology and Radiological Sciences, Division of Nuclear Medicine and Molecular Imaging, Johns Hopkins School of Medicine, Baltimore, MD USA

## Abstract

**Aim/Introduction:**

[^18^F]FDOPA PET/CT is one of the most frequently used functional imaging modalities for the diagnosis of pheochromocytoma and paraganglioma (PPGL). The biochemical secretion type of PPGL is crucial for patient management, being linked to varying aggressiveness and metastatic risk. The aim of this study is to compare the biochemical phenotype and secretion with uptake intensity and tumour volume on [^18^F]FDOPA PET/CT.

**Methods:**

All patients with sympathetic PPGL undergoing [^18^F]FDOPA PET/CT and laboratory analysis at first diagnosis at LMU University Hospital between 03/2012 and 11/2023 were included. Metabolic tumour volume (MTV), SUVmax, SUVmean and total lesion uptake (TLU) were compared to biochemical secretion using Rho-Spearman’s correlation and linear regression correcting for age, gender and secretion type. Biochemical phenotypes were compared with Mann-Whitney-U-test, ROC analysis was used to test the diagnostic discriminative power of radioligand uptake.

**Results:**

71 of 74 PPGL were [^18^F]DOPA-positive. TLU and MTV showed a moderate to strong correlation with plasma and urinary normetanephrines (*R* = 0.68–0.82, *p* < 0.001), plasma 3-methoxytyramine (*R* = 0.50, *p* = 0.003) and urinary metanephrines (*R* = 0.69–0.80, *p* < 0.001). Regression analysis revealed a significant relationship between biochemical secretion and TLU (r^2^ = 0.45–0.52, *p* < 0.001). Compared to adrenergic PPGL, noradrenergic PPGL demonstrated an increased radioligand uptake (*p* < 0.001). ROC analysis identified thresholds for SUVmax ( > 12.1) and SUVmean ( > 6.95) that moderately distinguished both phenotypes (AUC = 0.75-0.76).

**Conclusion:**

Radioligand uptake on [^18^F ]FDOPA PET/CT is associated with the biochemical phenotype of PPGL. This finding may facilitate the metabolic profiling of patients with suspected impaired or delayed laboratory results. Normetanephrine concentrations in plasma and 24-hour urine may be employed as predictive markers of MTV and TLU.

## Introduction

Pheochromocytoma (PHEO) and paraganglioma (PGL), collectively referred to as PPGL, arise from the sympathetic and parasympathetic parts of the autonomic nervous system. The sympathetic axis includes the adrenal medulla and chromaffin cells located in the posterior mediastinum or retroperitoneum, while parasympathetic paraganglia develop mainly in the head and neck region and in the anterior and middle mediastinum. Sympathetic PPGL are characterised by increased secretion of the catecholamines norepinephrine (NE) and epinephrine (E) which is associated with their clinical hallmarks of tachycardia, palpitations, pallor, tremor and hyperhidrosis (Eisenhofer et al. 2011a).

Quantification of the catecholamine metabolites normetanephrine (NMN), metanephrine (MN) and 3-methoxytyramine (3-MT) in plasma and urine allows for a sensitive and accurate diagnosis when preanalytical caveats are taken into account and appropriate assays are used (Eisenhofer et al. 2011). The measurement of catecholamine metabolites in plasma has been demonstrated to be a more effective diagnostic tool than urine measurement (Eisenhofer et al. 2018). Based on their secretion profile, PPGL are further subclassified as adrenergic, noradrenergic or dopaminergic phenotypes. This distinction has clinical implications, as the type of secretion influences symptoms, metastatic risk, tumor growth and may provide clues to the genotype (Eisenhofer et al. 2011a). PPGL have the highest degree of heritability of any tumour (Nölting et al. 2022). The germline mutations can be divided into three main molecular clusters. Cluster 1 comprises, amongst others, pathogenic variations in the SDHX and VHL genes, which result in tumour development through the stimulation of the HIF1α-signaling pathway. Cluster 2 encompasses amongst others pathogenic variations in the RET-, NF1-, or MAX-genes, resulting in tumour development via overactivation of the tyrosine kinase. Finally, Cluster 3, a rare occurrence, is distinguished by a disruption of the Wnt-signaling pathway.

Noradrenergic PPGL express less phenylethanolamine N-methyl transferase (PNMT), an enzyme catalysing the conversion of NE into E (Eisenhofer et al. 2011, 2008). A disruption of the secretory regulatory mechanisms causes a more continuous release of NE (Geroula et al. 2019). Additionally, the noradrenergic phenotype is associated with a higher tumor growth rate, metastatic potential and the genetic Cluster 1 (Nölting et al. 2022; Sarkadi et al. 2022a). In contrast, the adrenergic phenotype is primarily associated with the genetic Cluster 2 and characterised by a slower growth rate and a lower risk of metastasis (Eisenhofer et al. 2011). The largely intact regulatory mechanisms of catecholamine release result in a lower basal release with intermittent increases, while maintaining responsiveness to stimuli (Eisenhofer et al. 2008).

Positron-Emission-Tomography/Computed Tomography (PET/CT) imaging with 6-[^18^F]-L-fluoro-3,4-dihydroxyphenylalanine ([^18^F]FDOPA) is recommended at first diagnosis of PHEOs larger than five centimeters. For extra-adrenal PGL of any size and for suspected metastatic PPGL, it is recommended as a second-choice imaging method after somatostatin receptor targeted PET/CT (Taïeb et al. 2019; Casey et al. 2024; Timmers et al. 2024). [^18^F]FDOPA undergoes the biochemical transformations of endogenous L-DOPA, a precursor in catecholamine biosynthesis, after uptake by chromaffine cells with the L-type amino acid transporter 1 (LAT1) (Barollo et al. 2016). In comparison with healthy adrenal tissue, PPGLs display elevated LAT1 expression, leading to augmented [^18^F]FDOPA uptake (Manso et al. 2022).

In smaller cohorts, the concentration of catecholamine metabolites has been shown to correlate with metabolic tumour volume (MTV) and total lesion uptake (TLU) of PHEOs on [^18^F]FDOPA PET/CT (Amodru et al. 2018; Moog et al. 2018). The aim of the present study was to verify whether this correlation persists in a larger cohort. Additionally, the study aimed to investigate whether the quantitative PET parameters allow conclusions to be drawn about the biological phenotype of the tumours.

## Patients and methods

### Patient population

This retrospective study was conducted in the Departments of Nuclear Medicine and Internal Medicine IV at LMU University Hospital in Munich, Germany. All patients with PPGL who were examined by [^18^F]FDOPA PET/CT at initial diagnosis between 03/2012 and 11/2023 were included. Patients with a head-and-neck or ventral mediastinum PPGL were excluded, as these PPGL typically are hormonally inactive (Constantinescu et al. 2022). The collection of retrospective data was conducted in accordance with the Declaration of Helsinki. A local ethics vote from the institutional review board of the LMU University Hospital was available for the retrospective evaluation of the patient data (23–0689).

### PET/CT protocol and image reconstruction

Imaging was conducted according in clinical routine with commercially available [^18^F]FDOPA (IASOdopa®, IASON GmbH). A GE Discovery 690 PET/CT (General Electric) was used for whole-body PET/CT imaging in three-dimensional mode (3 min per bed position). The PET acquisition was started 60 minutes after intravenous application of 3 MBq [^18^F]FDOPA/kilogram body weight. Furosemide (20 mg) was applied immediately after injection of the radioligand to increase excretion unless there were medical contraindications (e.g., urolithiasis, urinary incontinence).

A diagnostic CT scan from the neck to the pelvis was acquired with automatic exposure control using tube current modulation (AutomA 3D; General Electric; scan parameters: 120 kV, 100–190 mAs, collimation 2 × 5 mm, 1.5). In addition, an iodine-based contrast agent (Ultravist 300™; Bayer Healthcare; 1.5 ml/kg body weight) was used to generate CT scans in the portal venous contrast phase. CT datasets were utilised for PET attenuation correction. PET images were reconstructed with a VPFX algorithm with a 6.5 mm gaussian filter.

### Image analysis

Metabolic tumour volume (MTV) of the primaries and metastatic lesions was determined through semi-automated segmentation with a maximum standard uptake value (SUVmax) threshold of 4.0 using the software Hermia Hybrid Viewer, Affinity 1.1.4, Hermes Medical Solutions, Stockholm, Sweden. The threshold was selected on the basis of preliminary research conducted by Noordzij et al. (Noordzij et al. 2019). SUVmax and the mean standard uptake value (SUVmean) of the MTV were assessed. Total lesion uptake (TLU) was calculated by multiplication of MTV and SUVmean. In patients with multiple primary tumours, the metabolic tumour volume (MTV) and total lesion uptake (TLU) of the individual lesions were summed to obtain a total value. Tumours with an SUVmax below 4.0 were rated [^18^F]FDOPA-negative; in these tumours, MTV and TLU were evaluated with 0 ml. SUVmean and SUVmax of the [^18^F]FDOPA-negative tumours was assessed manually.

### Laboratory analysis

The laboratory tests were included when conducted within three months prior to or after the PET/CT examination, but prior surgery. The concentration of O-methylated metabolites of catecholamines in plasma was determined using enzyme-linked immunosorbent assay (ELISA) and/or liquid chromatography-mass spectrometry (LCMS), according to the decision of the treating physician (Weismann et al. 2015). Laboratory values acquired with both methods were each compared separately to the radioligand uptake characteristics. Blood was drawn in the morning in a fasting state without prior intake of medications. Proton pump inhibitors were not paused during the days before blood drawing. Prior to blood sampling, patients were placed in supine position for a period of 20 minutes. The samples were immediately stored on ice until measurement if performed within 6 hours. Otherwise, the samples were stored at −20 °C.

The concentration of catecholamines and their O-methylated degradation products was determined in 24-hour urine samples by high-performance liquid chromatography.

The secretory phenotype was ascertained through the analysis of plasma concentrations of O-methylated catecholamine metabolites. The adrenergic biochemical phenotype was defined by an increased plasma concentration of MN above 62 pg/ml (0.31 nmol/L) and a tumour-related increase in MN of more than 5% of the combined increase in NMN and MN (Eisenhofer et al. 2011a). All other tumours were classified as noradrenergic tumours. Serum chromogranin A (CgA) was determined by ELISA (Cisbio) according to the manufacturer’s instructions.

### Statistical analysis

Statistical analysis was performed using the software SPSS (version 29.0.0.0 (241), International Business Machines Corporation, USA). Catecholamine metabolite concentrations in plasma and 24-hour urine samples were correlated with PET/CT metabolic parameters using Spearman-Rho correlation. In instances where plasma concentrations fell below the detection limit, random values between zero and the corresponding detection limit were generated by a random number generator (Excel 2016 version 1808, Microsoft-Office-365, USA). To ascertain the predictive value of the laboratory values on the TLU, variables were log-transformed, and a stepwise forward multiple regression was conducted, considering the variables laboratory value, secretion type, age and gender. The normality of the log-transformed variables was assessed using the Shapiro-Wilk test, and homoscedasticity was tested using the White-test. Mann-Whitney-U-test (MWU-test) was utilized to evaluate whether there was a significant difference of PET parameters of noradrenergic and adrenergic secretion types. To ascertain the extent to which SUVmax and SUVmean values could be suitable for differentiating adrenergic from noradrenergic PPGL, a ROC analysis was conducted. For determining statistical significance, an alpha level of 0.05 was used. Normally distributed numeric data are presented with mean ± standard deviation. Numeric data without Gaussian distribution are presented with median (interquartile range).

## Results

### Patient characteristics

77 patients underwent an [^18^F]FDOPA PET/CT as part of their initial diagnostic workup. Three patients were excluded due to a primary in the head-neck region or in the anterior mediastinum. The diagnosis of PHEO and PGL was confirmed histopathologically in 70 of the 74 cases included in the study. In the remaining cases an interdisciplinary tumour board based on clinical assessment, laboratory findings, as well as radiological and molecular imaging confirmed the diagnosis. At the time of initial diagnosis, two patients had been found to have lymphogenic metastases, 72 patients presented with non-metastatic disease. One patient with a germline mutation in the VHL-gene (Cluster 1B), who could not be assigned to a secretion type due to missing blood values, presented with bilateral pheochromocytoma and one lymphogenic metastasis. This metastasis was not detected by either the PET or CT components of the [^18^F]FDOPA PET/CT scan and was identified solely through histopathological examination. Another patient with a germline mutation in the SDHB gene (Cluster 1A) and a noradrenergic secretion phenotype exhibited one lymph node metastasis with mildly increased [^18^F]FDOPA uptake (SUVmax 3.1) and an additional lymph node metastasis that was not visible in either the PET or CT components of the [^18^F]FDOPA PET/CT. The latter metastasis was also identified histopathologically. Table 1 illustrates the distribution of patients according to gender, age, type of PPGL (PHEO/PGL) and plasma concentration of catecholamine metabolites. Information about genetic testing for predisposing germline pathogenic variants was available for 59 of the 74 patients, identifying 30 cases with a germline pathogenic variant favouring the development of PPGL. 29 of the 59 tested patients showed sporadic PPGL manifestations. In 10 of these 29 patients, somatic mutations were detected: six tumours with a somatic mutation in the NF1-gene, and two patients each with mutations in the EPAS1- and the VHL-gene. Median reported Ki67 index was 2% (1% − 3%), indicating a low proliferation rate. Within the cohort, catecholamine metabolite concentrations in blood were available for 70 patients. 26 patients exhibited a noradrenergic secretion type and 44 patients presented with an adrenergic secretion type. Catecholamine metabolites were quantified by LCMS in 34 patients, by ELISA in 58 patients and by both methods in 23 patients.

**Table 1:** Patient characteristics

Characteristics						n	median (IQR)	ULN
Gender	Female					46 (62.1%)		
	Male					28 (37.8%)		
Age [years]						46 ± 18.1		
Entity	PHEO					64 (86.4%)		
	PGL					6 (8.1%)		
	PPGL					4 (5.4%)		
Genetic variant	not available					15 (20.3%)		
	no pathogenic variant					19 (25.7%)		
	Cluster 1A					9 (12.2%)		
			SDHB		GM	4 (5.4%)		
			SDHC		GM	1 (1.4%)		
			SDHD		GM	4 (5.4%)		
	Cluster 1B					8 (10.8%)		
			VHL		GM	4 (5.4%)		
					SM	2 (2.7%)		
			EPAS1		SM	2 (2.7%)		
	Cluster 2					23 (31.1%)		
			NF1		GM	4 (5.4%)		
					SM	6 (8.1%)		
			MAX		GM	2 (2.7%)		
			RET		GM	11 (14.9%)		
Secretion type	noradrenergic					26		
	adrenergic					44		
Ki67 index						32 (43.2%)	2 (1% − 3%)	
ELISA	Metanephrine (pg/ml)					57 (77.0%)	79.0 (37.5 - 469.0)	< 100
	Normetanephrine (pg/ml)					58 (78.4%)	645.0 (185.0 - 1191.3)	< 216
LCMS	Metanephrine (ng/l)					34 (45.9%)	135.5 (64.5 - 327.3)	< 90
	Normetanephrine (ng/l)					34 (45.9%)	504.5 (233.5 - 2002.5)	< 200
	3-methoxytyramine (ng/l)					33 (44.6%)	12.0 (3.0 - 27.5)	< 28
PET-parameter	SUVmean					74 (100%)	6.4 (5.0 - 8.6)	
	SUVmax					74 (100%)	12.1 (7.4 - 18.7)	
	MTV					74 (100%)	13.3 (4.9 - 42.1)	
	TLU					74 (100%)	92.3 (25.0 - 342.3)	

Summary of patient characteristics, including gender, age, ethnicity ⦋pheochromocytoma (PHEO), paraganglioma (PGL)⦌, germline mutation (GM) and somatic mutation (SM), the proliferation index (Ki67), and plasma levels of catecholamine metabolites, which have been quantified by liquid chromatography-mass spectrometry (LCMS) and/or enzyme-linked immunosorbent assay (ELISA). The data are presented as median and interquartile range (IQR). ULN = Upper reference limit

### Radioligand uptake of PPGL on ^18^FDOPA PET/CT

PET scans were initiated 62 ± 11.8 minutes after injection of a median dose of 228.6 MBq (Q1: 204 MBq, Q3: 252.5 MBq) [^18^F]FDOPA. 71/74 patients (95.9%) showed visibly strong [^18^F]FDOPA uptake. Three patients showed a SUVmax below 4.0 and were therefore considered “[^18^F]FDOPA negative”. Two patients showed DOPA-negative PHEOs. Both patients were assigned to Cluster 2 due to the RET mutation (MEN 2A) and had an adrenergic secretion type. The third DOPA-negative patient had an SDHB germline mutation in abdominal paraganglioma and a noradrenergic type of secretion. In total, the median SUVmean of all included PPGL was 6.4 (Q1: 5.0, Q3: 8.6), the median SUVmax was 12.1 (Q1: 7.4, Q3: 18.7), the median MTV 13.3 ml (Q1: 4.9 ml, Q3: 42.1 ml) and the median TLU 92.3 (Q1: 25.0, Q3: 342.3).

### Correlation of biochemical secretion and metabolic tumour volume/total lesion uptake

Rho-Spearman’s correlation analysis showed a moderate to strong correlation between MTV and TLU on [^18^F]FDOPA PET/CT and different biochemical parameters quantified in plasma and 24-hour urine samples (see Table 2). Comparing laboratory measurements from plasma samples, there was a strong correlation between NMN and MTV/TLU (*R* = 0.708 - 0.793, *p* < 0.001) and a moderate correlation between 3-MT with MTV/TLU (*R* = 0.504, *p* = 0.003). Plasma MN did not show a significant correlation with MTV or TLU. In 24-hour collection urine, there was a strong correlation between MTV, TLU and absolute NMN and MN levels (*R* = 0.678 - 0.820, *p* < 0.001), a moderate correlation with NE levels (*R* = 0.462 - 0.619, *p* < 0.001) and a weak correlation with absolute dopamine levels (*R* = 0.313, *p* = 0.046). The plasma concentration of chromogranin A correlated significantly with the metabolic volume of the PPGL (*R* = 0.472, *p* = 0.027), but not with the TLU (*R* = 0.326, *p* = 0.139).

**Table 2:** Spearman´s correlation of catecholamine metabolites and quantitative parameters on [^18^F]FDOPA-PET/CT

Spearman´s correlation		Total lesion uptake			Metabolic tumour volume		
		R	p	n	R	p	n
Metanephrine	LCMS (plasma)	−0.002	0.992	34	0.155	0.381	34
	ELISA (plasma)	0.015	0.915	57	0.069	0.608	57
	*a* (24 h urine)	**0.747**	** < 0.001**	39	**0.802**	** < 0.001**	40
	*c* (24 h urine)	**0.687**	** < 0.001**	46	**0.747**	** < 0.001**	46
Normetanephrine	LCMS (plasma)	**0.776**	** < 0.001**	34	**0.793**	** < 0.001**	34
	ELISA (plasma)	**0.708**	** < 0.001**	58	**0.744**	** < 0.001**	58
	*a* (24 h urine)	**0.801**	** < 0.001**	41	**0.820**	** < 0.001**	41
	*c* (24 h urine)	**0.680**	** < 0.001**	46	**0.678**	** < 0.001**	46
3-methoxytyramine	LCMS (plasma)	**0.504**	**0.003**	32	**0.504**	**0.003**	33
Epinephrine	*a* (24 h urine)	−0.087	0.592	40	−0.004	0.982	40
	*c* (24 h urine)	−0.137	0.353	48	−0.062	0.678	48
Norepinephrine	*a* (24 h urine)	**0.619**	** < 0.001**	41	**0.609**	** < 0.001**	41
	*c* (24 h urine)	**0.503**	** < 0.001**	48	**0.462**	** < 0.001**	48
Dopamin	*a* (24 h urine)	0.286	0.070	41	**0.313**	**0.046**	41
	*c* (24 h urine)	0.106	0.473	48	0.097	0.512	48
Chromogranin A	ELISA (plasma)	0.326	0.139	22	**0.472**	**0.027**	**22**

Spearman correlation coefficients (R) and corresponding p-values (p) for the relationships between total lesion uptake and metabolic tumour volume and laboratory parameters in plasma and 24-hour urine are shown. Plasma catecholamine metabolite concentrations were quantified by ELISA and/or LCMS. Both the absolute amount of catecholamine metabolites (a) and the concentration of each catecholamine metabolite in the 24-hour urinary samples (c) were considered. Significant correlation coefficients are highlighted in bold

### Prediction of metabolic tumour volume with biochemical secretion

**Fig. 1:**
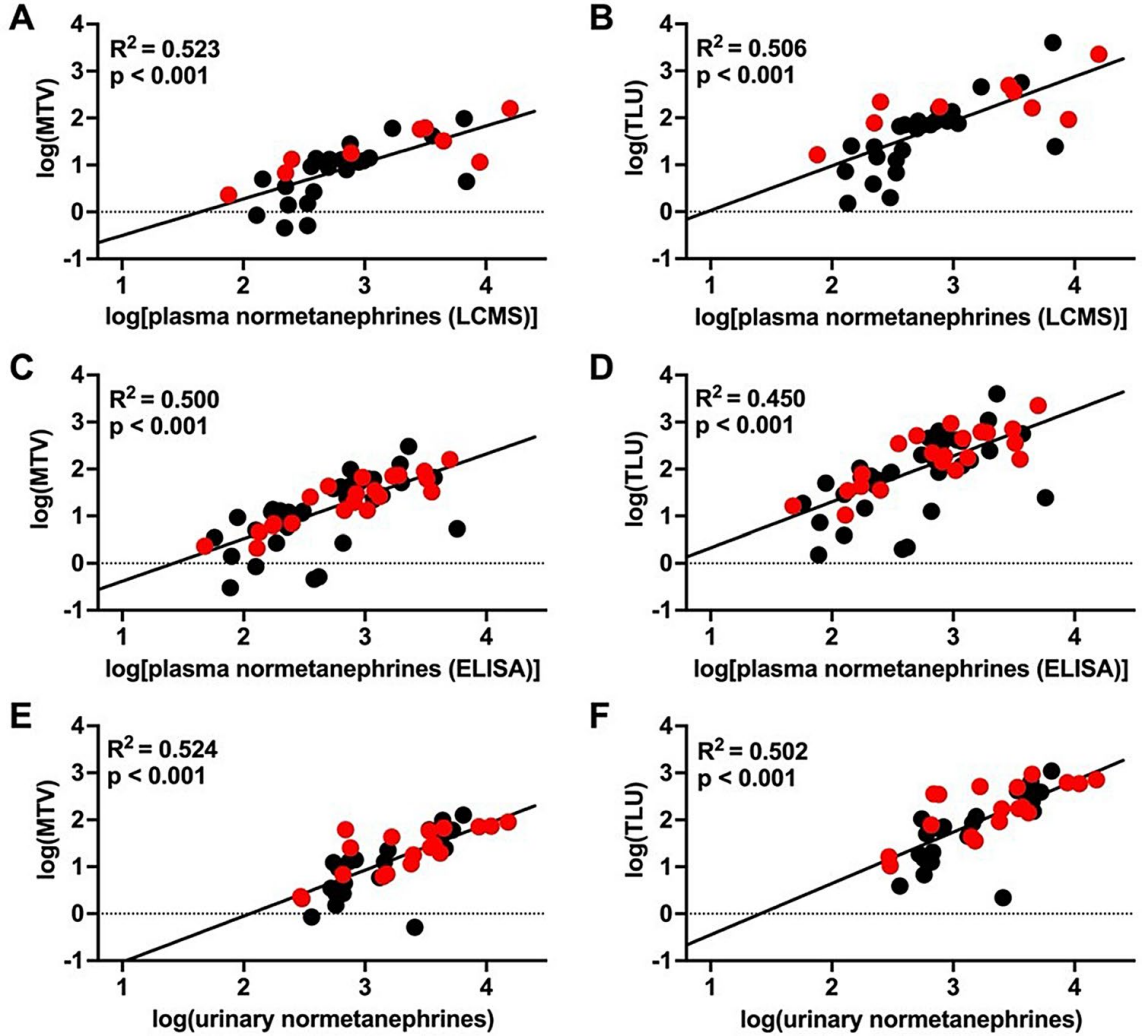
Prediction of total lesion uptake (TLU) and metabolic tumour volume (MTV) with normetanephrine levels measured in plasma and urine using linear regression. The logarithmically transformed hormone values (X-axis) are plotted against the logarithmically transformed TLU and MTV (Y-axis) in a scatter plot (noradrenergic = red, adrenergic = black). Adjusted R^2^ and p-values of the regression model are displayed in the graphs. Plasma normetanephrine concentrations, as determined by either LCMS (Figure **A**, **B**) or ELISA (Figure **C**, **D**), in conjunction with 24-hour urinary levels (Figure **E**, **F**)

Following the establishment of a significant correlation between laboratory parameters and MTV and TLU, a stepwise multiple regression was conducted, with the log transformed analyte concentrations as independent variables. In instances where age, sex or secretion type had a significant effect on the model, these were incorporated as independent covariables. NMN in plasma and 24-hour urine significantly predicted MTV and TLU. Age, gender, and secretion type did not exert a significant effect on MTV and TLU. The results of the analysis are presented in Table 3 and in Fig. 1.

**Table 3:** Prediction of metabolic tumour volume (MTV) and total lesion uptake (TLU) with plasma and urinary levels of normetanephrine using stepwise multiple regression. Plasma values were measured with LCMS and ELISA

	Predictor variable				Whole model			
	Rc	SE	ß-value	p-value		Adusted R^2^	F-value	p-value
**Prediction of MTV**								
NMN LCMS	**0.85**	**0.14**	**0.73**	** < 0.001**		**0.52**	**36.09**	** < 0.001**
NMN ELISA	**0.90**	**0.12**	**0.71**	** < 0.001**		**0.50**	**56.07**	** < 0.001**
NMN (24h urine)	**0.98**	**0.15**	**0.73**	** < 0.001**		**0.52**	**43.92**	** < 0.001**
**Prediction of TLU**								
NMN LCMS	**0.95**	**0.16**	**0.72**	** < 0.001**		**0.51**	**33.73**	** < 0.001**
NMN ELISA	**0.97**	**0.14**	**0.68**	** < 0.001**		**0.45**	**45.93**	** < 0.001**
NMN (24h urine)	**1.09**	**0.17**	**0.72**	** < 0.001**		**0.50**	**40.33**	** < 0.001**

### Image parameters of PPGL with different biochemical secretion type

**Fig. 2:**
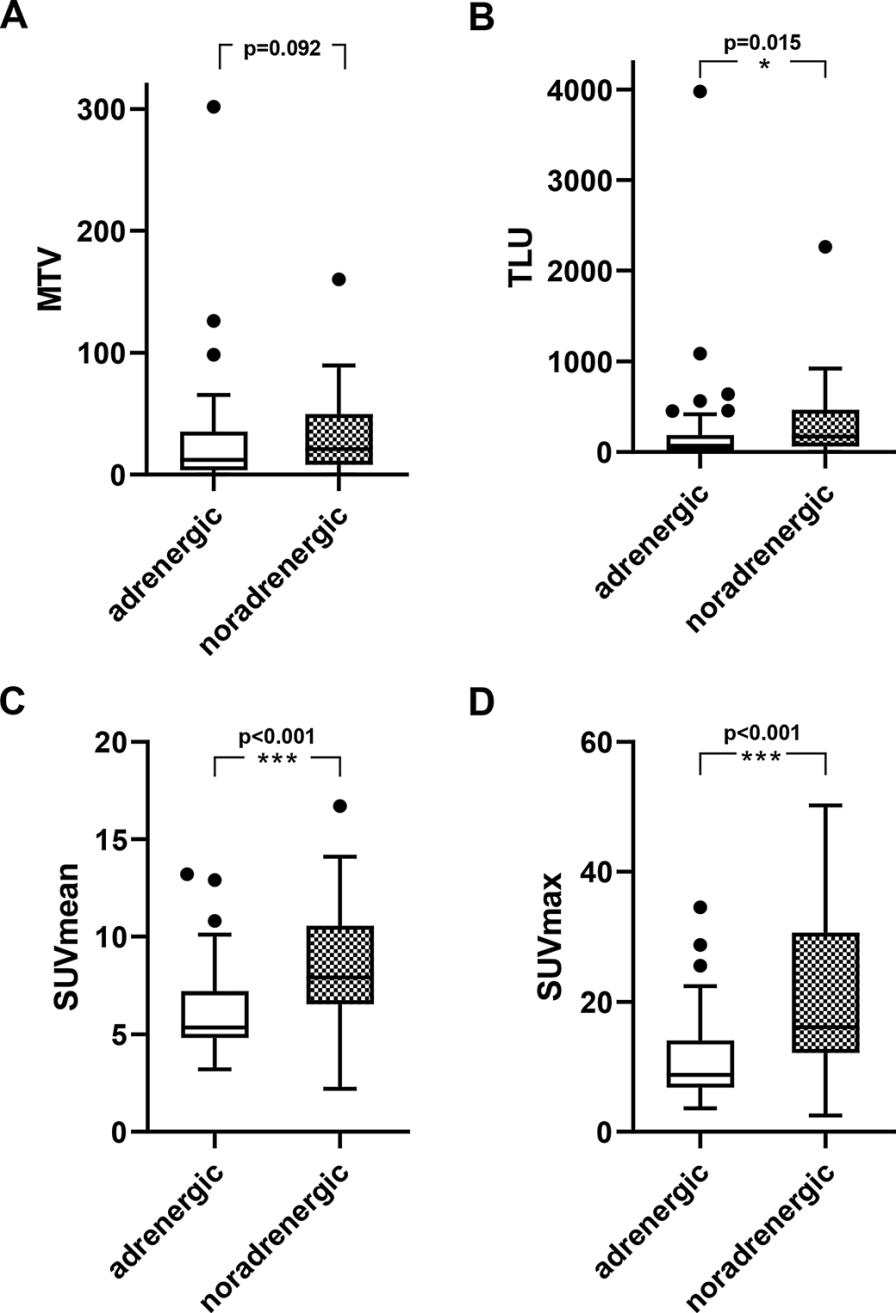
Image parameters of adrenergic and noradrenergic PPGL on [^18^F]FDOPA-PET. Metabolic tumour volume (MTV, **A**) was not significantly increased in noradrenergic PPGL, total lesion uptake (TLU, **B**), SUVmean (**C**) and SUVmax (**D**) are significantly increased in PPGL of the noradrenergic phenotype. For the classification, no differentiation was not made between the dopaminergic and the noradrenergic phenotype

**Fig. 3:**
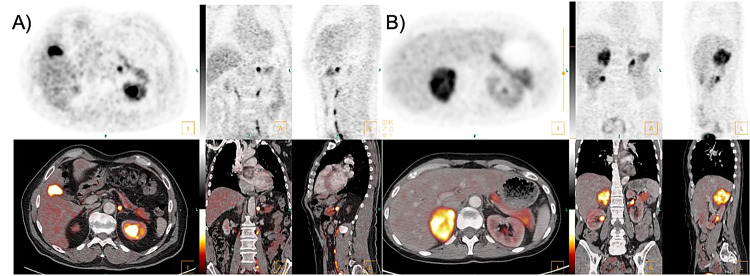
Patient examples with adrenergic and noradrenergic phenotype. (**A**): [^18^F]FDOPA PET/CT of a 65-year-old male patient with a small left-sided pheochromocytoma with strong radioligand uptake. The adrenal mass was detected incidentally on a CT scan performed due to suspected urolithiasis. The patient had a two-year history of intermittent vertigo and tremor. The levels of metanephrine (67 ng/l) and 3-methoxytyramine ( < 10 ng/l) were within the normal range, the normetanephrine concentration was slightly elevated at 206 ng/l (norm < 200 ng/l). [^18^F]FDOPA PET/CT showed increased [^18^F]FDOPA uptake (SUVmax 14.7, SUVmean 7.1, MTV 2.3 ml, TLU 16.4 ml*SUV). The patient declined genetic testing. (**B**): [^18^F]FDOPA PET/CT of a 53-year-old female patient with right-sided pheochromocytoma with a typical rim-enhancement and central cystic parts. The patient presented for the differential diagnosis of an adrenal incidentaloma. Clinically, the patient presented with palpitations and an arterial hypertension. While the metanephrine concentration of 60 ng/l was within the normal range, a significantly elevated normetanephrine concentration of 3190 ng/l (norm < 200 ng/l) was found. 3-methoxytyramine was moderately elevated at 43.0 ng/l. Significantly elevated levels of chromogranin A (844 µg/l) were detected. On [^18^F]FDOPA-PET/CT, the mass showed increased radioligand uptake (SUVmax 9.6, SUVmean 5.8, MTV 61.1 ml, TLU 354.4 ml*SUV). Genetic analysis showed no evidence of a hereditary origin

The biochemical secretion type can be assessed based on the assessment of plasma hormone levels. Our data showed a [^18^F]FDOPA-positive imaging in 25/26 patients with noradrenergic phenotype (96.2%) and in 42/44 (95.5%) patients with the adrenergic phenotype. Noradrenergic PPGL showed significantly higher SUVmax and SUVmean than adrenergic tumours (*p* < 0.001), see also Fig. 2. The MTV of noradrenergic tumours was slightly larger than the MTV of adrenergic PPGL without reaching statistical significance (*p* = 0.092). The median TLU within the adrenergic group was significantly lower (*p* = 0.015) than that of the noradrenergic group (68.3 (19.0 - 188.0) SUV*ml vs. 171.0 (68.6 - 497.8) SUV*ml). Table 4 provides an overview of the median and interquartile range (Q1 and Q3) of plasmatic catecholamine metabolites, [^18^F]FDOPA uptake (SUVmean, SUVmax, TLU) and tumour size (MTV) for both groups. [^18^F]FDOPA PET/CT examples of patients with noradrenergic and adrenergic phenotype are presented in Fig. 3.

**Table 4:** Image parameters of adrenergic and noradrenergic secretion types

	adrenergic (*n* = 44)	noradrenergic (*n* = 26)	p-values
ELISA Metanephrine (pg/ml) Normetanephrine (pg/ml)	278.0 (93.5–762.0) 414.0 (179.5–1001.0)	36.0 (25.5–41.0) 844.0 (215.0–1788.5)	
LCMS Metanephrine (ng/l) Normetanephrine (ng/l) 3-methoxytyramine (ng/l)	169.5 (107.3–717.8) 451.0 (252.5–955.3) 12.0 (3–29.0)	49.5 (35.0–61.5) 1827.0 (186.8–5536.8) 14.5 (6.8–27.3)	
SUVmax	8.8 (6.8–14.1)	16.0 (12.2–31.2)	< 0.001
SUVmean	5.4 (4.8–7.2)	7.9 (6.6–11.5)	< 0.001
MTV [ml]	12.1 (3.6–35.3)	22.5 (6.9–58.1)	0.092
TLU	68.3 (19.0–188.0)	171.0 (68.6–497.8)	0.015

The [^18^F]FDOPA storage parameters of both groups, including metabolic tumour volume (MTV), maximum standardised uptake value (SUVmax), mean standardised uptake value (SUVmean) and total lesion uptake (TLU), are presented in tabular form. Quantification of plasma catecholamine metabolites was conducted using liquid chromatography-mass spectrometry (LCMS, *n* = 34) and/or enzyme-linked immunosorbent assay (ELISA, *n* = 58). The data are presented as medians with interquartile ranges

### Radioligand uptake-based ROC analysis for biochemical subtyping

**Fig. 4:**
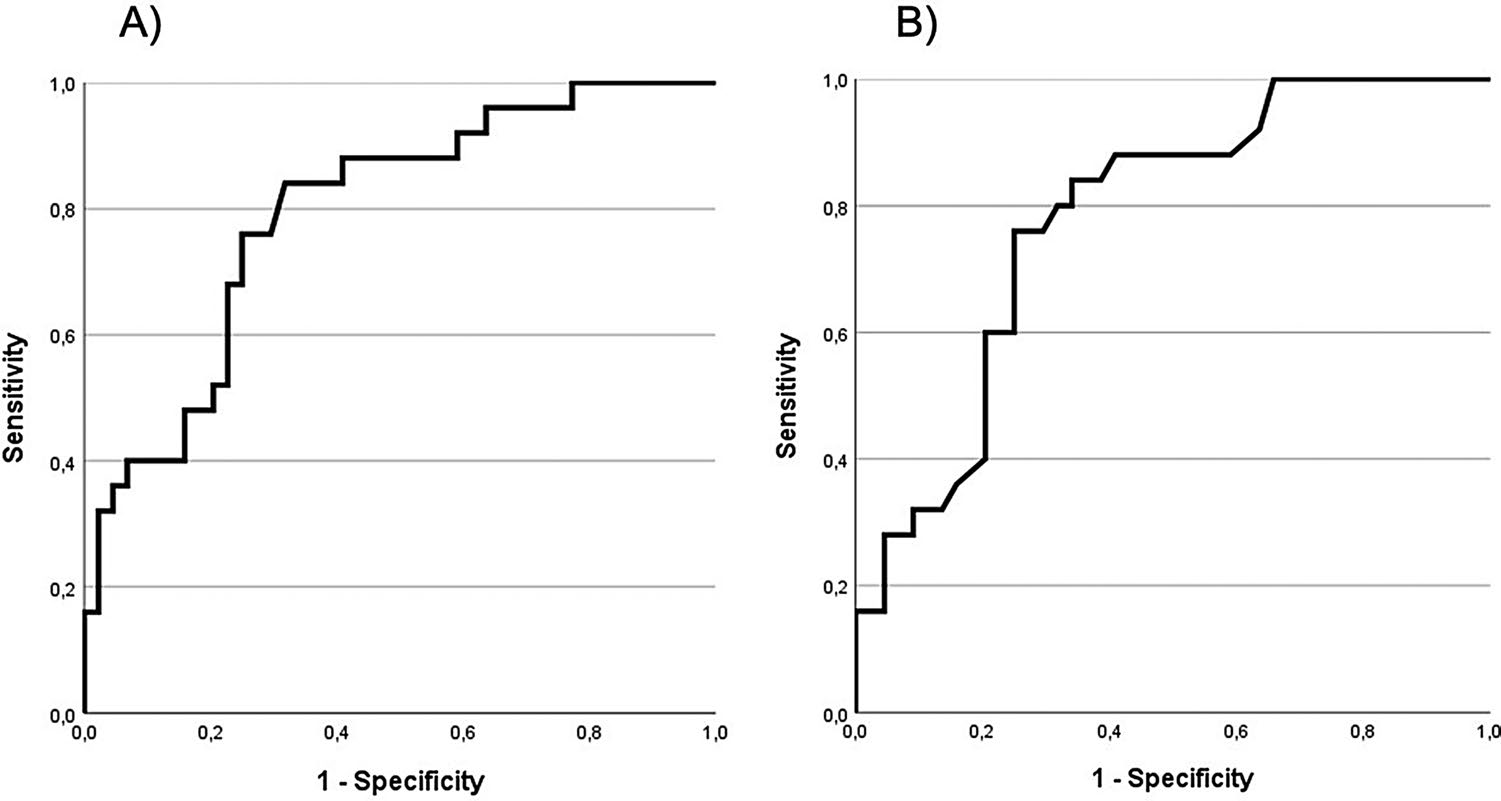
Differentiation of the biochemical phenotype based on radioligand uptake on [^18^F]FDOPA-PET/CT. The curve demonstrates the efficacy of SUVmax (**A**), AUC of 0.763 and SUVmean (**B**), AUC of 0.749 in differentiating between secretion types based on the higher [^18^F]FDOPA uptake observed in noradrenergic PPGL (*n* = 26) compared to adrenergic PPGL (*n* = 44). An optimal discrimination was reached at a cut-off of SUVmax 12.1 (sensitivity 81%, specificity 68%) and SUVmean of 6.95 (sensitivity 73%, specificity 75%)

A receiver operating characteristic (ROC) analysis and determination of the Youden index were conducted to differentiate PPGL into adrenergic (*n* = 44/70) and noradrenergic (*n* = 26/70) secretion types based on the intensity of their radioligand uptake (see Fig. 4). The area under the curve (AUC) was 0.763 (SUVmax) and 0.749 (SUVmean), indicating a moderate discriminatory ability. The optimal cut-off value for SUVmax was 12.1 with a sensitivity for the presence of a noradrenergic secretion type of 80.8%, a specificity of 68.2% and a Youden index of 0.490. The optimal cut-off value for SUVmean was 6.95 with a sensitivity for the presence of a noradrenergic secretion type of 73.1%, a specificity of 75.0% and a Youden index of 0.481.

## Discussion

Estimation of aggressiveness and metastatic potential remains a critical challenge in the diagnostic and therapeutic management of PPGL. Variations in malignancy are, among other factors such as genetic predisposition, linked to the biochemical phenotype. This study demonstrates a robust correlation between catecholamine secretion and imaging parameters on [^18^F]FDOPA PET/CT supporting the value of molecular imaging as a complementary tool to laboratory analysis in the characterization of PPGL. Noradrenergic PPGL showed a significantly enhanced radioligand uptake on [^18^F]FDOPA PET/CT. An ROC analysis revealed a moderate discriminatory power of [^18^F]FDOPA PET/CT. This distinction could be used as an additional information in the classification of PPGL subtypes.

Our study found that the radioligand uptake (SUVmean, SUVmax and TLU) was increased in noradrenergic PPGL in comparison with adrenergic PPGL. The sensitivity in our mostly non-metastasised cohort was excellent for both adrenergic and noradrenergic PPGL. These results demonstrate, that [^18^F]FDOPA is useful for the detection of adrenergic and noradrenergic non-metastasized PPGL. A receiver operating characteristic (ROC) analysis enabled the estimation of the biological phenotype based on the [^18^F]FDOPA radioligand uptake with a SUVmax threshold of 12.1 or a SUVmean threshold of 6.95. The capacity to estimate the secretion type based on [^18^F]FDOPA uptake is of clinical relevance considering that the measurement of catecholamine metabolites can be affected by a multitude of factors. For instance, through the collection of blood samples without prior resting of the patient, through prior administration of specific pharmaceuticals as well as caffeine or nicotine intake prior to the blood sampling (Schürfeld et al. 2024; Därr et al. 2014; Nölting et al. 2019). Additionally, this finding is of particular importance, as the secretion type provides valuable information regarding tumour aggressiveness, and metastatic risk which should be considered in the postoperative patient management (Nölting et al. 2022; Sarkadi et al. 2022).

Among the analyzed hormones, the strongest correlation with MTV and TLU was observed with NMN in urine and plasma. Additionally, a lower, but still moderate, correlation was found between urinary NE and MTV and TLU. A previous study examined the relationship between radioligand uptake on [^18^F]FDOPA PET/CT and catecholamine metabolites in a smaller cohort of 39 patients with PHEO (Moog et al. 2018). This previous study similarly found that metabolic tumour volume correlated with 24 h urinary normetanephrines (*r* = 0.64, *p* < 0.0001, *n* = 36) and plasma free normetanephrines (*r* = 0.55, *p* = 0.006, *n* = 23). Our findings and the previous literature suggest that the NMN in plasma and the NMN in urine could be used as an orientative surrogate parameter for the expected MTV and TLU, irrespective of age, sex and secretion type.

Our data also show a moderate correlation between MTV and TLU and 3-MT levels in plasma. 3-MT is elevated not only in dopaminergic PPGL, but also in approximately two thirds of the PPGL associated with SDHB and SDHD mutations (Eisenhofer et al. 2011, 2011a, 2017). Conversely, no significant correlation was identified between TLU and dopamine in urine. This observation is consistent with the known fact that the measurement of dopamine in urine is inadequate for determining the tumour’s dopamine release given its lack of specificity (Brown and Allison 1981; Eisenhofer et al. 2011a, 2005).

While we observed a strong correlation between urinary MN with TLU and MTV, there was no significant correlation between plasma MN and MTV or TLU. Elevated MN are a characteristic feature of adrenergic PPGL secreting catecholamines in a pulsatile manner. The secreted epinephrine is rapidly converted into metanephrine and cleared from the body through urinary excretion (Dalal 2023; Gu et al. 1999). A 24-hour urine collection effectively captures this fluctuating release of the rapidly converted epinephrine (Eisenhofer et al. 2008). The correlation between urinary metanephrines and TLU corroborates results from Amodru et al. who analyzed DOPA-PET in 56 PPGL patients and found, that total lesion uptake and SUVmax correlated significantly with metanephrines in the 24-hour urine using a 42% isocontour of SUVmax of the PPGL for a semiautomatic segmentation of the MTV (Amodru et al. 2018). They also showed that TLU of PPGL correlated with the ratio of urinary normetanephrines and urinary metanephrines as a surrogate for the biochemical phenotype (Amodru et al. 2018). In conclusion, plasma levels of normetanephrines and urinary levels of metanephrines could be used as surrogate parameters for estimating the anticipated tumour volume.

Manso et al. demonstrated that LAT1-expression in pheochromocytomas correlates positively with SUVmean and 24h urinary norepinephrine, but not with epinephrine metabolites (Manso et al. 2022). This finding as well as a higher rate of total catecholamine secretion (Eisenhofer et al. 2011b) may explain the higher [^18^F]FDOPA uptake observed in noradrenergic PPGLs compared to adrenergic PPGLs in the present study.

A correlation between chromogranin A and the histologically determined tumour volume, PET-based MTV on [^18^F]FDOPA PET/CT has been documented previously (Amodru et al. 2018; Bílek et al. 2019). Our findings confirm these previous finding, showing a moderate correlation between chromogranin A and the PET-based MTV. However, the statistical significance was weaker compared to the correlation of catecholamine levels with the metabolic tumour volume. Chromogranin A can be affected by several factors like the intake of certain medications like proton pump inhibitors or steroids and comorbidities like an impaired renal function (Kanakis and Kaltsas 2012). These confounding factors may account for the reduced statistical strength observed in the chromogranin A-MTV correlation.

This study is limited by its retrospective design and a small, predominantly PHEO-based cohort. For several patients, not all laboratory data were available. Future multicenter studies are necessary to compare the radioligand uptake in patients with different locations of primaries and with different genetic subtypes.

The present study has demonstrated that an augmented [^18^F]FDOPA uptake is associated with a noradrenergic secretion type, which is regarded as a risk factor for metastasis, irrespective of SDHx mutation status. Therefore, [^18^F]FDOPA PET/CT uptake could serve as an additional parameter for risk stratification by helping to identify more aggressive tumour histotypes, supporting its role as a complementary imaging biomarker in PPGL characterization.

## Data Availability

The datasets used and/or analyzed during the current study are available from the corresponding author upon reasonable request.
